# TWEAK regulates the functions of hair follicle stem cells via the Fn14‐Wnt/β‐catenin‐CXCR4 signalling axis

**DOI:** 10.1111/wrr.70032

**Published:** 2025-05-05

**Authors:** Xiaoyan Zou, Yaning Tian, Lingling Peng, Mai Luo, Zhu Yan, Zihan Xue, Xiaoming Liu, Yumin Xia

**Affiliations:** ^1^ Department of Dermatology The Second Affiliated Hospital of Xi'an Jiaotong University Xi'an China; ^2^ Department of Dermatology The First Affiliated Hospital of Xi'an Medical University Xi'an China; ^3^ Core Research Laboratory The Second Affiliated Hospital of Xi'an Jiaotong University Xi'an China; ^4^ Department of Dermatology Southern University of Science and Technology Hospital Shenzhen China

**Keywords:** CXCR4, Fn14, hair follicle stem cell, TNFR, TWEAK

## Abstract

Hair follicle stem cells (HFSCs) are crucial for maintaining cutaneous functions under various pathological conditions, including wounds. Tumour necrosis factor‐like weak inducer of apoptosis (TWEAK) interacts with its receptor, fibroblast growth factor‐inducible 14 (Fn14), and plays a role in the development and tissue repair of skin diseases. This study aims to elucidate the effects of TWEAK/Fn14 signalling on HFSCs and the associated mechanisms. The expressions of HFSC markers, including K19, integrin β1 and K15, were analysed via immunohistochemistry in normal and Fn14‐deficient mouse skin. Primary HFSCs were cultured in vitro and then treated with TWEAK or a chemokine (C—X—C motif) (CXCR) 4 inhibitor. The phenotype markers and secreted cytokines of HFSCs were assessed via immunofluorescence analysis, Western blotting and real‐time polymerase chain reaction. Our results showed that both Fn14 and CXCR4 were highly expressed in hair follicles. Fn14 deficiency led to a decrease in the expression levels of K19 and CD34. Exogenous TWEAK enhanced the expression of K15, K19, integrin β1, tumour necrosis factor receptor type 2 and CXCR4 in cultured HFSCs. Additionally, TWEAK induced the proliferation, migration and cytokine production in HFSCs. Furthermore, the Wnt/β‐catenin signalling pathway was upregulated in HFSCs upon TWEAK stimulation, and inhibitors of β‐catenin or CXCR4 suppressed the effects of TWEAK on the differentiation and secretory functions of HFSCs. In conclusion, TWEAK/Fn14 interaction regulates the expression of differentiation markers and secretory functions of HFSCs in vitro. Wnt/β‐catenin signalling or CXCR4 activation mediates the effects of TWEAK on HFSCs. Targeting the Fn14‐Wnt/β‐catenin‐CXCR4 signalling axis may offer a potential approach for managing HFSC‐related skin diseases, such as wounds.

**Abbreviations**
CXCR4: chemokine (C—X—C motif) receptor 4
Fn14: fibroblast growth factor‐inducible 14
HFSC: hair follicle stem cell
TNFR: tumour necrosis factor receptor
TWEAK: tumour necrosis factor‐like weak inducer of apoptosis

## 1 INTRODUCTION

Hair follicle stem cells (HFSCs) are mainly located in the bulge region of hair follicles and are characterised by markers such as keratins 15 and 19 (K15/19), integrin β1, CD34 and Lgr5.^1^, ^2^, ^3^ HFSCs are also present in the follicular infundibulum, where they express markers like Lrig1 and Lgr6, and may migrate upward during the re‐epithelialisation process in skin wound repair.^4^ Recent studies have highlighted the crucial role of HFSCs in maintaining cutaneous functions under various pathological conditions.^5^, ^6^, ^7^ The depletion or quiescence of the HFSC pool contributes to the development of alopecia, and the activation of HFSCs is essential for drug‐induced hair regrowth.^5^ Narrow‐band ultraviolet B therapy induces repigmentation in human vitiligo skin through the activation of the beta‐catenin pathway in HFSCs.^6^ Additionally, Wnt/beta‐catenin signalling‐mediated HFSC regeneration plays a significant role in promoting wound healing in the skin.^7^ HFSCs promote cutaneous wound healing through the stromal cell‐derived factor 1alpha/C—X—C motif chemokine receptor (CXCR) 4 axis.^8^ Furthermore, HFSCs are primarily responsible for hair regeneration and give rise to skin epithelium for tissue repair under injury.^9^ Thus, HFSCs are crucial for maintaining various functions of skin tissue.

Tumour necrosis factor‐like weak inducer of apoptosis (TWEAK) is a proinflammatory factor that regulates the functions of skin resident cells by interacting with its receptor, fibroblast growth factor‐inducible 14 (Fn14). Both TWEAK and Fn14 are weakly expressed in normal skin tissue but are significantly upregulated in lesional skin.^10^, ^11^, ^12^ TWEAK levels are elevated in the sera of patients with severe alopecia.^13^ Fn14 signalling mediates UVB‐induced Ro52 expression and photosensitization in cutaneous lupus erythematosus.^10^ Moreover, the TWEAK/Fn14 signalling pathway plays a role in the development of atopic dermatitis and psoriasis.^7^, ^12^ Activation of the TWEAK/Fn14 signals enhances the migration and cytokine production of dermal microvascular endothelial cells and dermal fibroblasts, which contribute to burn wound repair.^14^ Therefore, TWEAK is a crucial regulator of skin tissue renewal and repair.

Recent studies suggest that TWEAK/Fn14 signalling has pleiotropic functions in regulating various stem cell fates.^15^ TWEAK influences the proliferation, differentiation and migration of multiple stem and progenitor cells located in the liver, brain, bone, muscle, adipose tissue and other organs.^15^ For example, TWEAK promotes the expression of inhibitor of differentiation‐1 in hepatic progenitor cells, leading to aberrant differentiation of these cells.^16^ Moreover, Fn14‐mediated signalling positively regulates myoblast fusion and skeletal muscle regeneration through the activation of the Wnt signalling pathway.^17^ Additionally, the expressions of stem‐like phenotype markers are enhanced in ovarian cancer cells upon the stimulation of TWEAK in combination with a chemotherapeutic agent (carboplatin).^17^ Conversely, blocking the TWEAK‐Fn14‐RelB signalling cascade extends survival following carboplatin chemotherapy in a mouse model of ovarian cancer.^18^ TWEAK induces the differentiation of endothelial progenitor cells into mature endothelial cells and promotes their migration to the wound area, thereby accelerating wound healing.^15^ These findings underscore the crucial role of TWEAK in the biological functions of various stem and progenitor cells. This study aims to elucidate the effects of TWEAK/Fn14 signalling on HFSCs and the associated mechanisms.

## 2 MATERIALS AND METHODS

### 2.1 Haematoxylin–eosin staining and immunohistochemistry

Fn14 deficiency was induced in Balb/c mice using the CRISPR/Cas9 engineering approach.^14^ Both wild‐type and Fn14 knockout mice were housed at the animal facility of Xi'an Jiaotong University Medical School. Ten‐week‐old male mice were used in the experiments. As described previously,^7^ the mouse model of skin wound was constructed on the back of wild‐type mice. This study was conducted with the ethical approval (No. 2021‐1019) from the Ethical Committee of Xi'an Jiaotong University Medical School. The protocols used strictly followed the ARRIVE guidelines.

Fresh tissues were fixed in 4% paraformaldehyde and then embedded in paraffin using standard procedures. Haematoxylin and eosin staining was performed on deparaffinised sections. For immunohistochemistry analysis, sections were pretreated with 2% goat serum (Beyotime Biotech, Shanghai, China) and underwent antigen retrieval using sodium citrate buffer.^19^ Rabbit anti‐mouse K19 or rabbit anti‐mouse CD34 (Abcam, Cambridge, MA, USA) was used as the primary antibody at a concentration of 2 μg/mL. Polymer horseradish peroxidase‐labelled goat anti‐rabbit IgG (DAKO, Glostrup, Denmark) was used as the secondary antibody at a concentration of 2 mg/mL.

### 2.2 Cell culture and scratch assay

Human primary HFSCs were isolated from scalp samples as previously described.^20^ This protocol was approved by the Ethical Committee of Xi'an Jiaotong University Medical School (No. 2021‐1019). Briefly, the bulge of hair follicles, located between the isthmus and the upper part of the hair bulb, was separated using microscissors under a dissecting microscope (Leica, Wetzlar, Germany). HFSCs were then obtained through a two‐step proteolytic digestion and harvested using a differential enrichment procedure. The cells were cultured in a keratinocyte serum‐free medium (Thermo Fisher Scientific, Waltham, MA, USA) supplemented with Y‐27632 (MedChemExpress LLC, Princeton, NJ, USA). HFSCs were stimulated for 48 hours with recombinant human TWEAK (0–250 ng/mL; R&D Systems, Minneapolis, MN). The β‐catenin inhibitor XAV‐939 (MedChemExpress LLC) or the CXCR4 inhibitor endogenous peptide inhibitor for CXCR4 (EPI‐X4) (hSA408–423 peptide; MedChemExpress LLC) was added simultaneously to specific groups of HFSCs. These inhibitors were prepared in 0.1% dimethyl sulfoxide and phosphate buffered saline (PBS), respectively, with a final concentration of 1 μM in the culture supernatants.

To assess the migration ability of HFSCs, a standard scratch assay was performed as previously described.^21^ Cell monolayers were scratched with a pipette tip, and the resulting gaps were monitored at various time points using a digital camera‐equipped microscope.

### 2.3 Immunofluorescence analysis

Frozen sections were processed for immunofluorescence analysis as described previously.^14^ The sections were fixed in precooled (−20°C) acetone and then incubated with primary antibodies. After washing with PBS and incubation with 4',6‐diamidino‐2‐phenylindole (DAPI) solution (beyotime), the sections were examined using a fluorescence confocal microscope (Leica TCS SP2, Leica, Wetzlar, Germany). HFSCs cultured in glass‐bottom culture dishes (MatTek, Ashland, MA, USA) were fixed with 4% paraformaldehyde solution. They were then permeabilized with 0.2% Triton X‐100 and blocked with 2% goat serum (beyotime). The cells were incubated with primary antibodies at 4°C overnight, followed by DAPI staining and examination using confocal microscopy.^19^ The primary antibodies used in the immunofluorescence assay are listed in Table S1.

### 2.4 Flow cytometry

The 5‐ethynyl‐2′‐deoxyuridine (EdU) Cell Proliferation Kit with Alexa Fluor 488 (Beyotime) was used for flow cytometry. Cultured cells were routinely trypsinised and resuspended in a binding buffer. After incubation with EdU reagent for 2 h, cells were fixed with paraformaldehyde and permeabilised with Triton X‐100. Finally, cells were incubated with the EdU reaction reagent. Analysis was performed using the FACSAria II flow cytometer (BD Biosciences, San Diego, CA, USA), and data were analysed with FACSDiva 7.0 software (BD Biosciences).

### 2.5 Quantitative real‐time polymerase chain reaction

Total RNA was extracted from cell lysates using TRIzol reagent (Invitrogen, Carlsbad, CA, USA). cDNA was synthesised using a commercial reagent kit (Takara Bio, Kyoto, Japan). Quantitative real‐time PCR (qRT‐PCR) was then performed with SYBR Green Master Mix (Takara) and the ABI PRISM 7900HT Sequence Detection System (Applied Biosystems, Waltham, MA, USA). All PCR primers (Sangon Biotech, Shanghai, China) are listed in Table S2. The expression levels of target genes were calculated using the 2^−△△Ct^ method.

### 2.6 Western blotting

Protein lysates from cell cultures were extracted using radio‐immunoprecipitation assay lysis buffer supplemented with a protease inhibitor cocktail (Thermo Fisher Scientific). The protein extracts were separated by polyacrylamide gel electrophoresis and transferred to a polyvinylidene difluoride (PVDF) membrane (Millipore, Billerica, MA, USA). The membranes were incubated sequentially with rabbit primary antibodies (2 μg/mL; Abcam or Cell Signaling Technology Inc., Danvers, MA, USA) and horseradish peroxidase‐labelled goat anti‐rabbit IgG (2 μg/mL; Abcam). Protein expression was then detected using an electrochemiluminescence kit (Millipore). The primary antibodies used in this study are listed in Table S1. Band intensities were quantified using ImageJ version 1.54 software (National Institutes of Health, Bethesda, MD, USA).

### 2.7 Enzyme‐linked immunosorbent assay

Cytokine levels were measured in the supernatants of cell cultures using ELISA kits (Abcam). The kits were employed to detect EGF (Cat. #ab217772), transforming growth factor β1 (TGF‐β1; Cat. #ab100647), VEGF (Cat. #ab222510), bFGF (Cat. #ab99979) and NGF (Cat. #ab193760).

### 2.8 Statistical analysis

All quantitative data are presented as mean ± standard error of the mean (SEM). Statistical differences between control and treatment groups were assessed using one‐way analysis of variance or *t*‐tests. Statistical analysis was conducted using GraphPad Prism 8.01 software (GraphPad Software, Boston, MA, USA). A *p*‐value less than 0.05 was considered statistically significant.

## 3 RESULTS

### 3.1 TWEAK/Fn14 signalling upregulates the expression of HFSC phenotype markers

**FIGURE 1:**
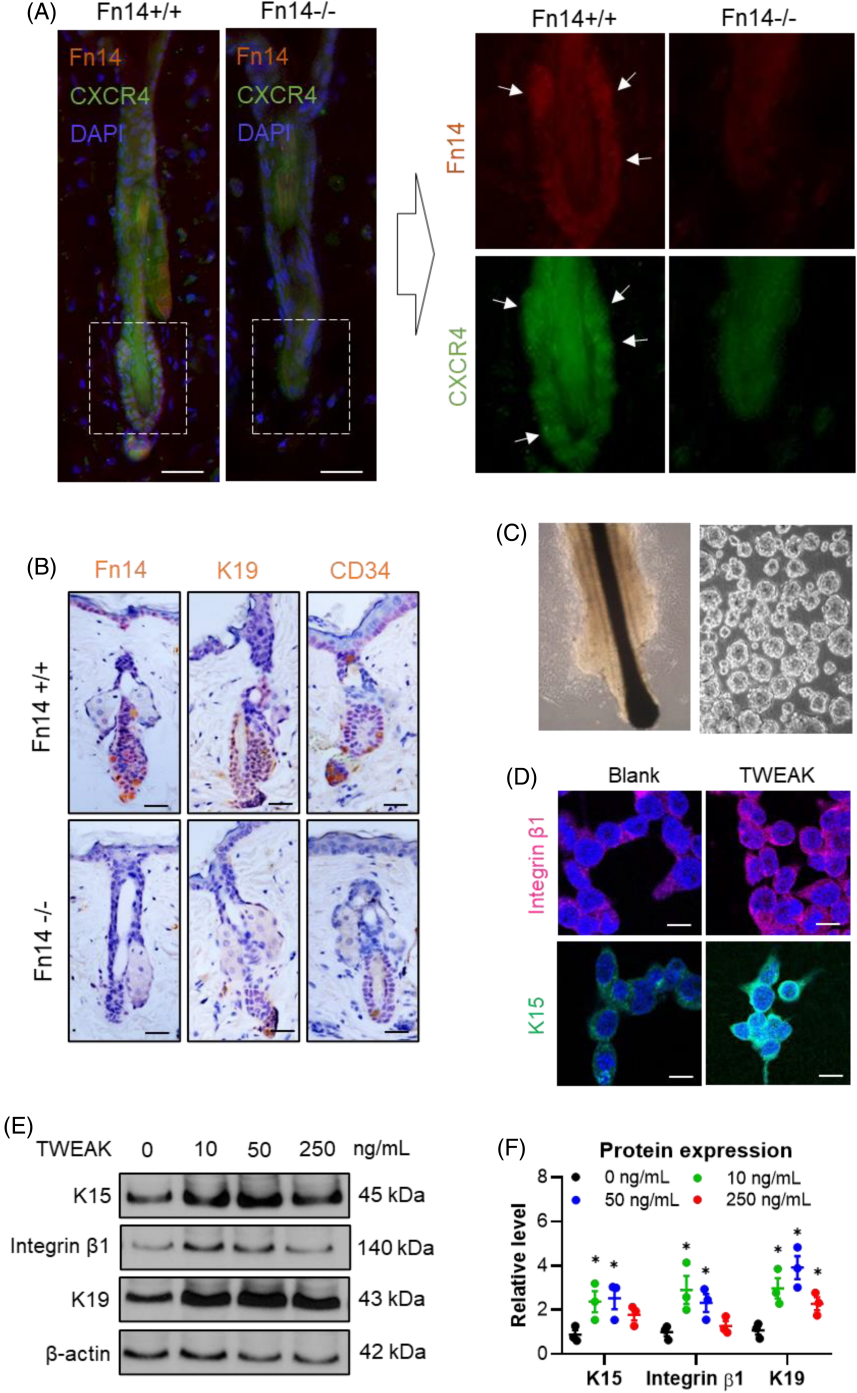
TWEAK/Fn14 signalling upregulates the expressions of HFSC markers. (A) Immunofluorescence analysis of Fn14 and CXCR4 expression in mouse hair follicles. The white arrows indicate the Fn14‐ or CXCR4‐positive cells; however, these positive cells were significantly diminished and even became indistinct in the Fn14‐deficient specimen. Scale bar = 20 μm. (B) Histochemical analysis of Fn14, K19 and CD34 expression in mouse hair follicles. Scale bar = 20 μm. (C) Primary human HFSCs cultured in vitro. (D) Immunofluorescence analysis of β1 integrin and K15 expression in HFSCs stimulated with TWEAK (10 ng/mL). Scale bar = 10 μm. (e, f) Western blotting of K19, integrin β1 and K15 proteins. Data represent mean ± SEM from three independent experiments. *p < 0.05, compared with the 0 ng/mL TWEAK group. CXCR4, chemokine (C—X—C motif) receptor 4; Fn14, fibroblast growth factor‐inducible 14; HFSC, hair follicle stem cells; K15/K19, keratins 15/19; TWEAK, tumour necrosis factor‐like weak inducer of apoptosis.

The effect of TWEAK/Fn14 signalling on HFSC markers was investigated in mouse skin tissue and cultured human HFSCs. Immunofluorescence analysis revealed that Fn14 and CXCR4 were highly co‐expressed in hair follicles (Figure 1A). Additionally, Fn14 was upregulated in the bulge of hair follicles, and Fn14 deficiency resulted in decreased expression of K19 and CD34 (Figure 1A,B). Primary human HFSCs were cultured in vitro (Figure 1C), and exogenous TWEAK increased the expression of both integrin β1 and K15 in the cells (Figure 1D). The upregulation of K19, integrin β1 and K15 proteins was confirmed by Western blotting (Figure 1E,F). These results indicate that Fn14 is associated with the overexpression of HFSC markers, and TWEAK promotes the upregulation of these markers.

TWEAK/Fn14 signalling mediates burn wound repair in mice,^14^ which is largely dependent on HFSC function.^22^ The expressions of TWEAK, Fn14 and HFSC markers were also assessed in lesional tissue from a mouse model of a skin wound. TWEAK was found to be diffusely expressed around hair follicles in the wounds. Additionally, the expressions of K15, integrin β1 and Lgr5 were elevated in these areas (see Figure S1). These results indicate that TWEAK/Fn14 molecules and HFSC markers fluctuate synchronously in response to wound inflammation.

### 3.2 TWEAK induces the proliferation, migration and cytokine production of HFSCs in vitro

**FIGURE 2:**
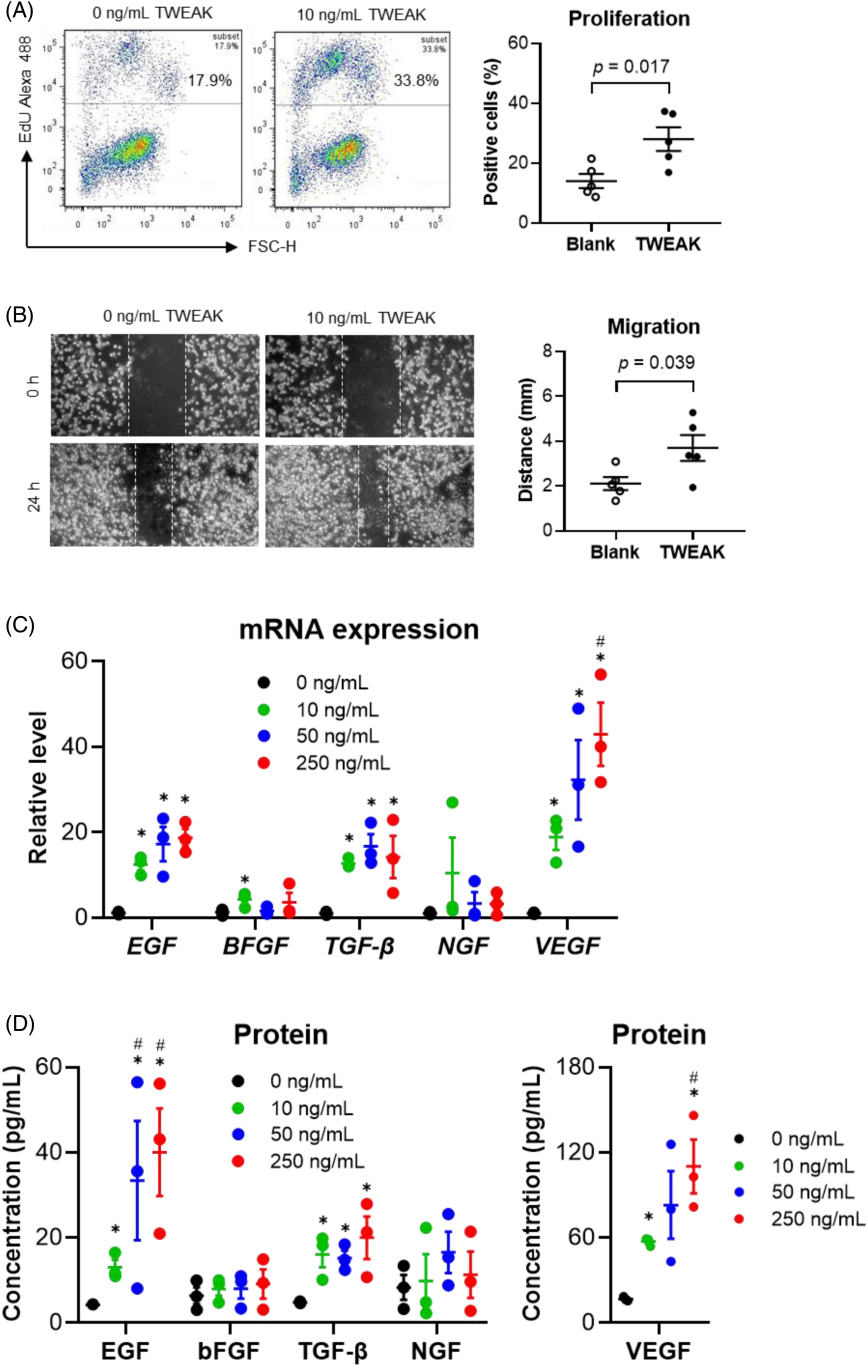
TWEAK induces proliferation, migration and cytokine production in HFSCs. Human HFSCs were cultured in vitro and stimulated with TWEAK (0–250 ng/mL). (A) Proliferation of HFSCs analysed via flow cytometry. *n* = 5 per group. (B) Cell migration assessed via scratch analysis. *n* = 5 per group. (C) mRNA expression levels of *EGF*, *BFGF*, *TGF‐β*, *NGF* and *VEGF* measured via qRT‐PCR. *n* = 3 per group. (D) Cytokine levels in the supernatants determined via ELISA. *n* = 3 per group. Data represent mean ± SEM from three to five independent experiments. *p < 0.05 compared with the 0 ng/mL group. #*p* < 0.05 compared with the 10 ng/mL group. HFSC, hair follicle stem cells; qRT‐PCR, quantitative real‐time PCR; SEM, standard error of the mean; TWEAK, tumour necrosis factor‐like weak inducer of apoptosis.

The biological effect of TWEAK on HFSCs was further analysed through in vitro experiments. TWEAK stimulation increased HFSC proliferation (Figure 2A). Additionally, scratch assay results indicated that TWEAK enhanced cell migration (Figure 2B). Therefore, TWEAK promotes both the proliferation and migration of HFSCs.

The secretion of cytokines relevant to wound healing is one of the intrinsic properties of HFSCs.^15^, ^23^ We analysed the production of several cytokines and found that exogenous TWEAK (0–250 ng/mL) increased the mRNA expression levels of *EGF*, *TGF‐β* and *VEGF*, while the expression of *BFGF* (coding bFGF) and *NGF* remained unchanged (Figure 2C). Similarly, the levels of EGF, TGF‐β and VEGF proteins were elevated in the supernatants (Figure 2D).

### 3.3 Fn14, TNFR2, IGFR and CXCR4 are upregulated in HFSCs upon TWEAK stimulation

**FIGURE 3:**
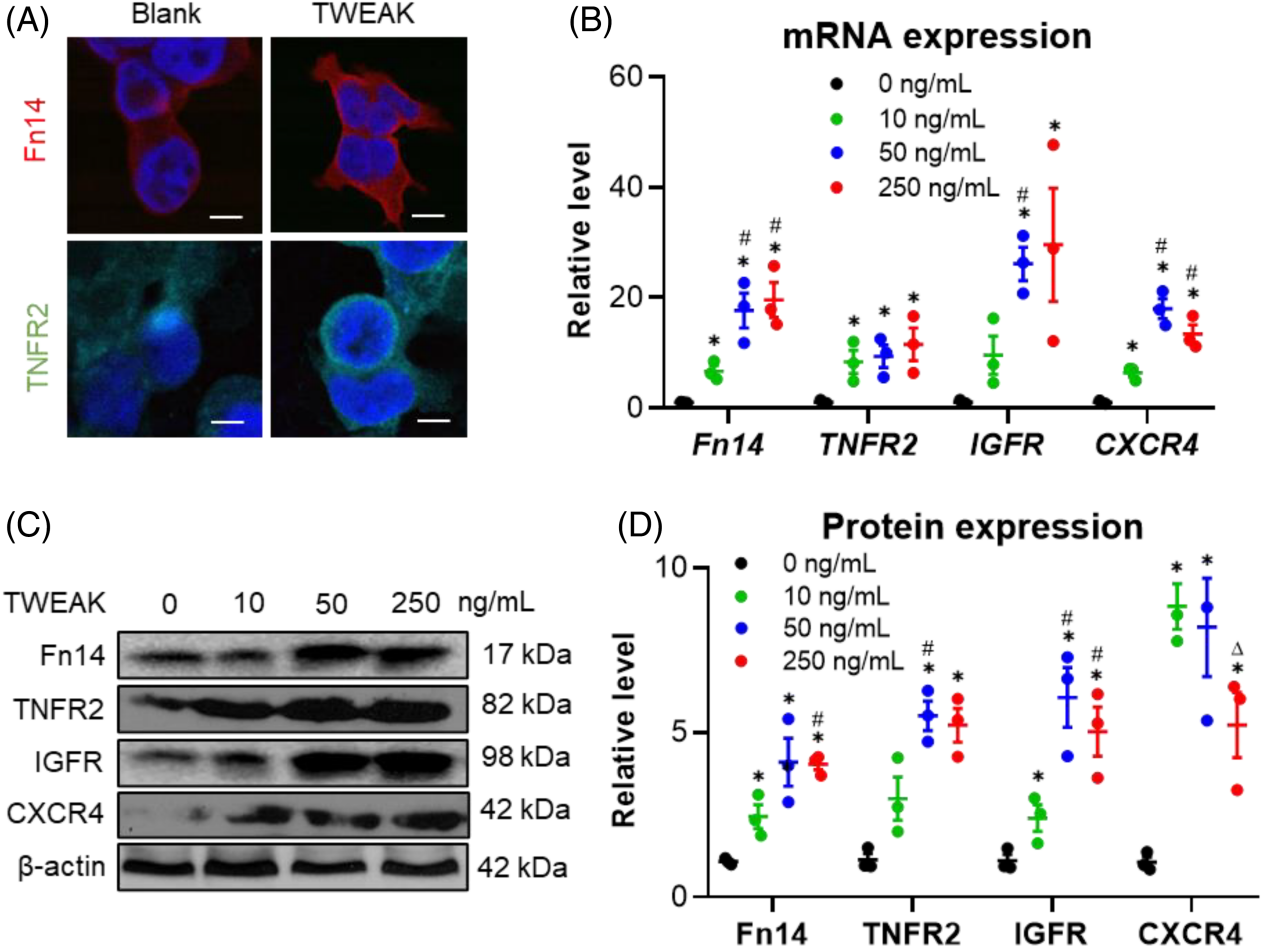
TWEAK upregulates the expressions of HFSC phenotype markers. Human HFSCs were cultured in vitro and stimulated with TWEAK (0–250 ng/mL). (A) Fn14 and TNFR2 expressions detected via immunofluorescence. (B) mRNA expression levels of *FN14*, *TNFR2*, *IGFR* and *CXCR4* analysed via qRT‐PCR. (C, D) Protein expressions of these markers determined via Western blotting, with quantification using ImageJ software. Data represent mean ± SEM from three independent experiments. *p < 0.05 compared with the 0 ng/mL group. #*p* < 0.05 compared with the 10 ng/mL group. Δ*p* < 0.05 compared with the 50 ng/mL group. HFSC, hair follicle stem cells; qRT‐PCR, quantitative real‐time PCR; SEM, standard error of the mean; TWEAK, tumour necrosis factor‐like weak inducer of apoptosis.

The protein expression profile of HFSCs may alter in response to certain environments.^24^ In vitro stimulation with exogenous TWEAK was used to investigate these changes. Immunofluorescence experiments revealed that both Fn14 and tumour necrosis factor receptor type 2 (TNFR2) were overexpressed following TWEAK stimulation (10 ng/mL) (Figure 3A). Additionally, mRNA expression levels of *FN14*, *TNFR2*, insulin‐like growth factor 1 receptor (*IGFR*) and *CXCR4* increased with TWEAK stimulation (0 to 250 ng/mL) (Figure 3B). This enhancement was further confirmed by Western blotting analysis (Figure 3C,D). Thus, TWEAK upregulates markers associated with the HFSC phenotype.

### 3.4 TWEAK activates Wnt/β‐catenin signalling in HFSCs in vitro

**FIGURE 4:**
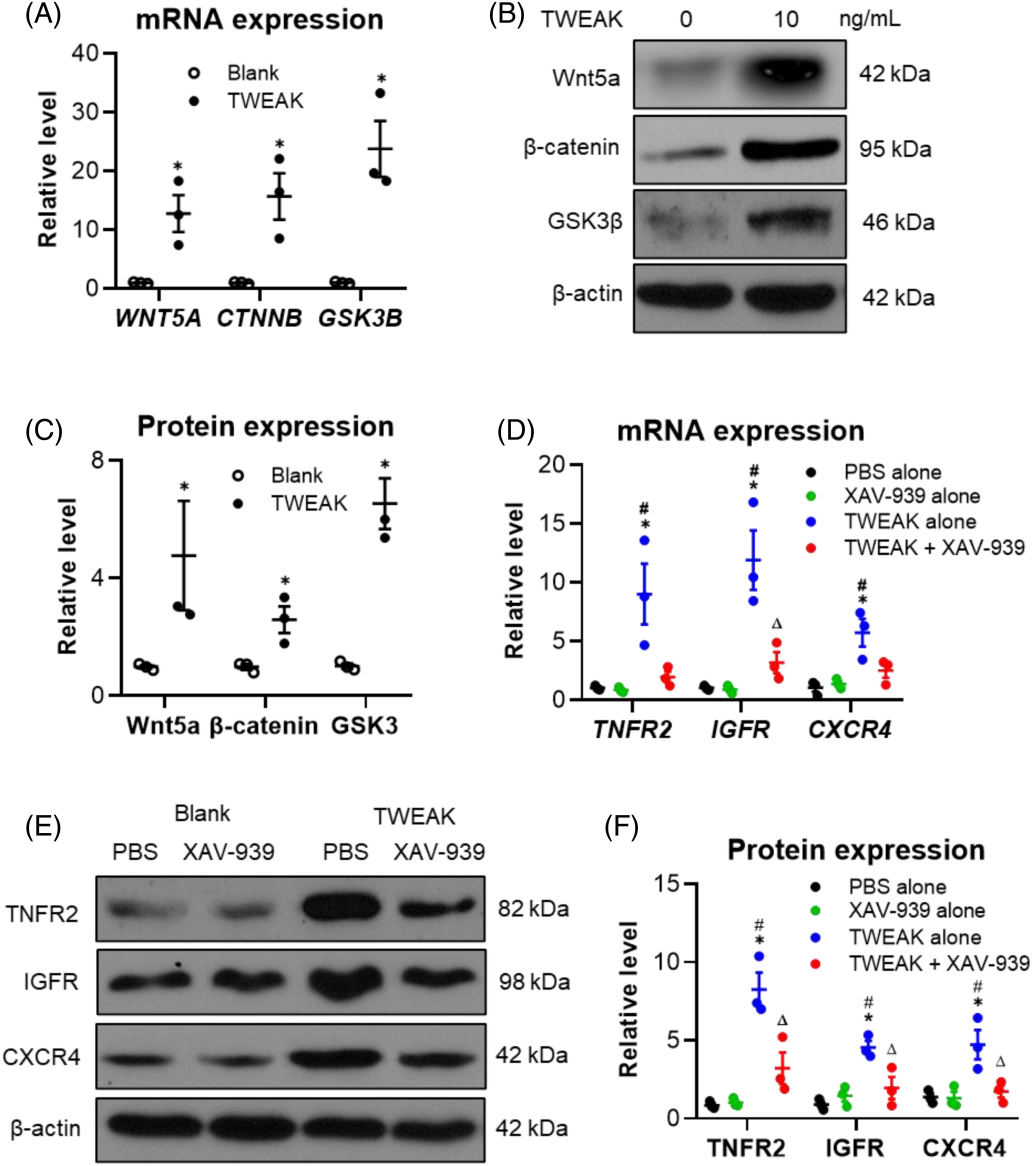
TWEAK activates Wnt/β‐catenin signalling in HFSCs. Human HFSCs were cultured in vitro and stimulated with TWEAK (10 ng/mL). (A) mRNA expression levels of *WNT5A*, *CTNNB* and *GSK3B* analysed via qRT‐PCR. (B, C) Protein expressions of these markers detected via Western blotting, with quantification using ImageJ software. (D) mRNA expression levels of *TNFR2*, *IGFR* and *CXCR4* analysed in cells pretreated with the β‐catenin inhibitor XAV‐939. (E, F) Protein expressions of these markers detected via Western blotting, with quantification using ImageJ software. Data represent mean ± SEM from three independent experiments. In (A, C), *p < 0.05 compared with the blank group. In (D, F), *p < 0.05 compared with the PBS alone group; #*p* < 0.05 compared with the XAV‐939 group. Δ*p* < 0.05 compared with the TWEAK alone group. HFSC, hair follicle stem cells; qRT‐PCR, quantitative real‐time PCR; TWEAK, tumour necrosis factor‐like weak inducer of apoptosis.

To further investigate the signalling pathways involved in TWEAK regulation, HFSCs were treated with TWEAK (10 ng/mL) in combination with the β‐catenin inhibitor XAV‐939. TWEAK alone increased the mRNA expression levels of *WNT5A*, *CTNNB* (which encodes β‐catenin) and glycogen synthase kinase 3β (*GSK3B*) (Figure 4A). Correspondingly, their protein levels were also upregulated by TWEAK (Figure 4B,C). Furthermore, TWEAK enhanced both mRNA and protein expressions of TNFR2, IGFR and CXCR4 in HFSCs, but this effect was blocked by XAV‐939 treatment (Figure 4D–F). Thus, the Wnt/β‐catenin signalling pathway mediates TWEAK regulation in HFSCs.

### 3.5 CXCR4 blockade inhibits the effect of TWEAK on HFSCs in vitro

**FIGURE 5:**
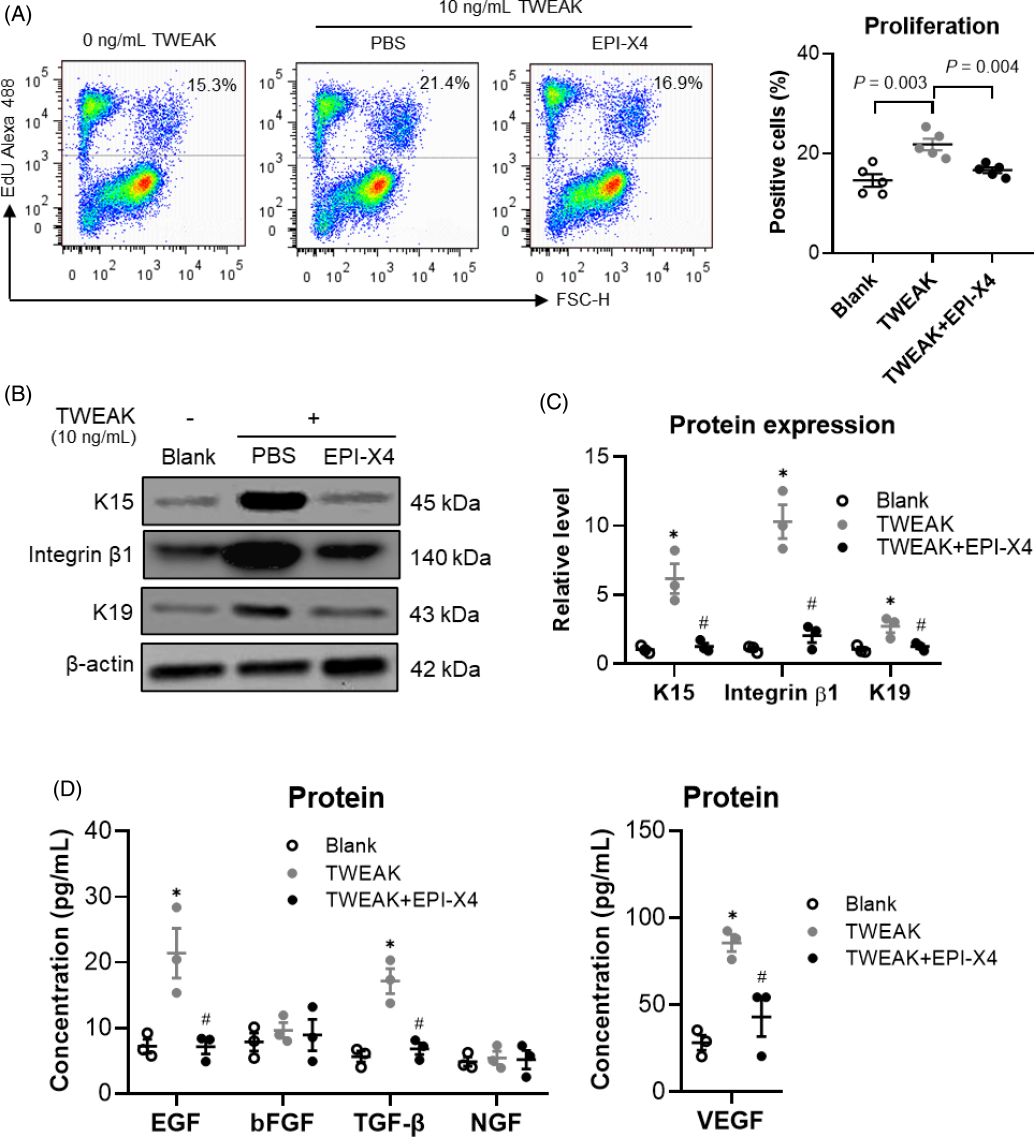
CXCR4 inhibitor suppresses the effect of TWEAK on HFSCs. Human HFSCs were cultured in vitro and treated with TWEAK (10 ng/mL) in the presence or absence of the CXCR4 inhibitor EPI‐X4. (A) Proliferation of HFSCs assessed via flow cytometry. *n* = 5 per group. (B, C) Protein expressions of differentiation markers detected via Western blotting and quantitated using ImageJ software. *n* = 3 per group. (D). Cytokines synthesised by HFSCs and measured via ELISA in the culture supernatants. *n* = 3 per group. Data represent mean ± SEM from three to five independent experiments. *p < 0.05 compared with the blank group. #*p* < 0.05 compared with the TWEAK alone group. CXCR4, chemokine (C—X—C motif) receptor 4; HFSC, hair follicle stem cells; TWEAK, tumour necrosis factor‐like weak inducer of apoptosis.

Considering that TWEAK upregulated the expression of TNFR2 and CXCR4 in HFSCs, we investigated the impact of TNFR2 or CXCR4 blockade on HFSC biological functions. Flow cytometry results showed that TWEAK stimulation (10 ng/mL) increased HFSC proliferation, but this effect was inhibited by the CXCR4 inhibitor EPI‐X4 (Figure 5A). Western blot analysis revealed that EPI‐X4 blocked the TWEAK‐induced increase in the protein expression of HFSC markers, including K15, integrin β1 and K19 (Figure 5B,C). These surface markers exhibit dynamic changes in expression levels within HFSCs, reflecting cellular differentiation states or distinct progenitor subpopulations.^1^, ^25^, ^26^ Additionally, EPI‐X4 treatment reduced the expression of cytokines, including EGF, TGF‐β and VEGF, while neither TWEAK nor EPI‐X4 affected the levels of bFGF and NGF (Figure 5D). Thus, CXCR4 blockade attenuates the effects of TWEAK on HFSCs.

## 4 DISCUSSION

In this study, we demonstrate that TWEAK not only upregulates HFSC markers but also enhances the proliferation, migration and cytokine production of HFSCs. Additionally, Fn14 signalling increases the levels of TNFR2, IGFR, CXCR4 and Wnt/β‐catenin molecules in HFSCs. Furthermore, blocking Wnt/β‐catenin or CXCR4 signalling inhibits the effects of TWEAK on HFSCs. Therefore, TWEAK regulates HFSC function through the Fn14–Wnt/β‐catenin–CXCR4 signalling axis.

Previous studies have shown that Fn14 is expressed in pluripotent stem cells in the liver, muscle, adipose tissue, brain and other tissues.^15^, ^16^, ^27^ TWEAK promotes the differentiation and proliferation of mesenchymal stem cells, hepatic progenitor cells and neural progenitor cells and enhances the migration of endothelial progenitor cells.^15^, ^16^, ^18^ Blocking the TWEAK–Fn14 interaction inhibits intestinal cell death induced by haematopoietic stem cell transplantation and reduces graft‐versus‐host disease.^28^ Our results align with these findings, demonstrating a similar regulatory role of TWEAK on HFSCs. However, we also found that TWEAK upregulates TNFR2 and IGFR and activates the Wnt/β‐catenin and CXCR4 signalling pathways. Additionally, TWEAK stimulates the production of EGF, TGF‐β and VEGF by HFSCs. Thus, the TWEAK/Fn14 interaction modulates the proliferation, differentiation and migration of stem cells by activating downstream signalling pathways and cytokines. These factors are crucial components of the stem cell niche and influence the fate of various stem cells.

The Janus kinase/signal transducer and activator of transcription (JAK–STAT), Wnt and Notch signalling pathways regulate the proliferation and differentiation of stem cells.^15^ We found that TWEAK increases the expression levels of Wnt5a, β‐catenin and GSK3β proteins, which are key components of the Wnt signalling pathway. Furthermore, the β‐catenin inhibitor XAV‐939 nullifies the regulatory effect of TWEAK on HFSCs. The Wnt/β‐catenin signalling pathway mediates the regulation of TWEAK in HFSCs. Wnt5a confers self‐renewal capacity in stem cells by inducing the expression of molecules related to the Notch pathway, while JAK–STAT signalling mediates the effects of Wnt signals.^29^ C—X—C motif chemokine ligand 12 binds to CXCR4 on cells, leading to increased nuclear localization of β‐catenin and a decrease in the expression of β‐catenin target genes.^29^, ^30^ Therefore, CXCR4 and β‐catenin engage in a reciprocal feedback loop: CXCR4 activation drives nuclear translocation of β‐catenin, which in turn transcriptionally upregulates CXCR4 expression. Targeting the Wnt/β‐catenin‐CXCR4 signalling axis could be a potential strategy for regulating the functions of stem cells, including HFSCs.

TNFR1 and TNFR2 are the two receptor types that interact with TNF‐α and TWEAK, and their activation triggers caspase‐8‐dependent cell death and canonical nuclear factor‐κB‐mediated cell proliferation.^31^ The predominance of TNFR1 or TNFR2 expression in resident cells depends on the local microenvironment. While TNFR2 is predominantly expressed in immune cells, it is also found in cancer cells as well as in psoriatic and human papillomavirus‐infected keratinocytes.^32^, ^33^ The TWEAK/Fn14 interaction promotes the proliferation and migration of cells that predominantly express TNFR2.^32^, ^33^ In this study, we found that TWEAK enhances the expression of TNFR2 and CXCR4 in HFSCs. Transmembrane TNF‐α upregulates CXCR4 expression in cells through TNFR2.^34^ Therefore, TWEAK regulates the biological functions of HFSCs via the TNFR2‐CXCR4 axis.

Insulin‐like growth factor 1 (IGF1) and its receptor IGFR play a crucial role in promoting glucose metabolism in cells. Hypoxia works in conjunction with endocrine signalling to support the self‐renewal, proliferation and migration of embryonic germline stem cells. Under hypoxic conditions, IGFR facilitates symmetric self‐renewal and migration of these cells through the hypoxia‐inducible factor‐Oct4/CXCR4 loop.^35^ Additionally, blocking autocrine IGF‐1 reduces the viability of mesenchymal stem cells by inhibiting the Akt/GSK3β signalling pathway.^36^ IGF1 promotes both proliferation and migration of bone marrow mesenchymal stem cells through the IGFR‐mediated extracellular signal‐regulated kinase 1/2 signalling pathway.^37^ Therefore, it can be inferred that IGFR plays a key role in the Wnt/β‐catenin‐CXCR4 signalling pathway, mediating the effects of TWEAK on HFSCs.

There were some limitations in this study, including the absence of in vivo data about the regulatory effect of TWEAK on HFSCs as well as the verification of TWEAK/Fn14 signalling blockade in an animal model. Future studies should be focused on the exploration of upstream regulation of TWEAK/Fn14 signals, intrinsic mechanism underlying Fn14‐CXCR4 signalling axis and potential inhibitors by the application of both in vitro and in vivo experimental approaches.

In conclusion, TWEAK/Fn14 activation regulates the differentiation of HFSCs and enhances their proliferation, migration and cytokine production. The Wnt/β‐catenin‐CXCR4 signalling axis mediates the effects of TWEAK on HFSC functions. Targeting the Fn14‐Wnt/β‐catenin‐CXCR4 signalling axis presents a promising approach for managing HFSC‐related skin diseases.

## AUTHOR CONTRIBUTIONS

**Xiaoyan Zou:** Conceptualization; investigation; methodology; formal analysis; writing—original draft. **Yaning Tian:** Investigation. **Lingling Peng:** Formal analysis; project administration. **Mai Luo:** Investigation. **Zhu Yan:** Investigation. **Zihan Xue:** Formal analysis. **Xiaoming Liu:** Conceptualization; resources; project administration; writing—review and editing. **Yumin Xia:** Conceptualization; writing—review and editing.

## CONFLICT OF INTEREST STATEMENT

The authors have declare no conflicts of interest.

## Supporting information


**Figure S1.** TWEAK, Fn14 and HFSC markers are highly expressed around cutaneous wounds in mice. (A) The expressions of K15, integrin β1 and Lgr5 were prominent in the wound or normal areas. (B) Both TWEAK and Fn14 were highly expressed in these areas with different patterns. Data are from three independent experiments. Representative images are shown. Scale bar = 30 μm.

**Table S1.** Antibodies used for immunohistochemistry or Western blotting.
**Table S2.** Primers used for qRT‐PCR analysis.

## Data Availability

All data are presented in the figures and Supporting Information. There are no data available in a public open access repository.

## References

[1] Purba TS , Haslam IS , Shahmalak A , Bhogal RK , Paus R . Mapping the expression of epithelial hair follicle stem cell‐related transcription factors LHX2 and SOX9 in the human hair follicle. Exp Dermatol. 2015;24(6):462‐467. doi:10.1111/exd.12700 25808706

[2] Ibrahim MR , Medhat W , El‐Fakahany H , Abdel‐Raouf H , Snyder EY . The developmental & molecular requirements for ensuring that human pluripotent stem cell‐derived hair follicle bulge stem cells have acquired competence for hair follicle generation following transplantation. Cell Transplant. 2021;30:9636897211014820. doi:10.1177/09636897211014820 34053245 PMC8182633

[3] Singh R . Basal cells in the epidermis and epidermal differentiation. Stem Cell Rev Rep. 2022;18(6):1883‐1891. doi:10.1007/s12015-021-10256-1 35080747

[4] Gong L , Xiao J , Li X , Li Y , Gao X , Xu X . IL‐36α promoted wound induced hair follicle neogenesis via hair follicle stem/progenitor cell proliferation. Front Cell Dev Biol. 2020;8:627. doi:10.3389/fcell.2020.00627 32984299 PMC7493638

[5] Wen L , Fan Z , Huang W , et al. Retinoic acid drives hair follicle stem cell activation via Wnt/β‐catenin signalling in androgenetic alopecia. J Eur Acad Dermatol Venereol. 2025;39(1):189‐201. doi:10.1111/jdv.20000 38629345 PMC11664453

[6] Goldstein NB , Koster MI , Jones KL , et al. Repigmentation of human vitiligo skin by NBUVB is controlled by transcription of GLI1 and activation of the beta‐catenin pathway in the hair follicle bulge stem cells. J Invest Dermatol. 2018;138(3):657‐668. doi:10.1016/j.jid.2017.09.040 29054607 PMC6135256

[7] BangHong J , YuKun W , Ao S , et al. Low‐level laser activates Wnt/β‐catenin signaling pathway‐promoting hair follicle stem cell regeneration and wound healing: upregulate the expression of key downstream gene Lef 1. Skin Res Technol. 2024;30(6):e13807. doi:10.1111/srt.13807 38887112 PMC11182782

[8] Yari A , Heidari F , Veijouye SJ , Nobakht M . Hair follicle stem cells promote cutaneous wound healing through the SDF‐1α/CXCR4 axis: an animal model. J Wound Care. 2020;29(9):526‐536. doi:10.12968/jowc.2020.29.9.526 32924817

[9] Ito M , Liu Y , Yang Z , et al. Stem cells in the hair follicle bulge contribute to wound repair but not to homeostasis of the epidermis. Nat Med. 2005;11(12):1351‐1354. doi:10.1038/nm1328 16288281

[10] Liu Y , Xu M , Min X , et al. TWEAK/Fn14 activation participates in Ro52‐mediated photosensitization in cutaneous lupus erythematosus. Front Immunol. 2017;8:651. doi:10.3389/fimmu.2017.00651 28620393 PMC5449764

[11] Liu Q , Wang H , Wang X , et al. Experimental atopic dermatitis is dependent on the TWEAK/Fn14 signaling pathway. Clin Exp Immunol. 2020;199(1):56‐67. doi:10.1111/cei.13373 31515807 PMC6904660

[12] Gupta RK , Gracias DT , Figueroa DS , et al. TWEAK functions with TNF and IL‐17 on keratinocytes and is a potential target for psoriasis therapy. Sci Immunol. 2021;6(65):eabi8823. doi:10.1126/sciimmunol.abi8823 34797693 PMC8756771

[13] Al Taweel AI , Hamed AM , Abdelrahman AMN , Hassan MNI . Tumor necrosis factor‐like weak inducer of apoptosis: a novel serum marker in patients with severe alopecia. Int J Trichol. 2019;11(3):113‐117. doi:10.4103/ijt.ijt_9_19 PMC6580803 31360039

[14] Liu J , Liu Y , Peng L , et al. TWEAK/Fn14 signals mediate burn wound repair. J Invest Dermatol. 2019;139(1):224‐234. doi:10.1016/j.jid.2018.05.036 30081003

[15] Wang S , Li L , Cook C , Zhang Y , Xia Y , Liu Y . A potential fate decision landscape of the TWEAK/Fn14 axis on stem and progenitor cells: a systematic review. Stem Cell Res Ther. 2022;13(1):270. doi:10.1186/s13287-022-02930-z 35729659 PMC9210594

[16] Liu W , Gao L , Hou X , et al. TWEAK signaling‐induced ID1 expression drives malignant transformation of hepatic progenitor cells during hepatocarcinogenesis. Adv Sci. 2023;10(18):e2300350. doi:10.1002/advs.202300350 PMC10288241 37085918

[17] Tomaz da Silva M , Joshi AS , Castillo MB , et al. Fn14 promotes myoblast fusion during regenerative myogenesis. Life Sci Alliance. 2023;6(12):e202302312. doi:10.26508/lsa.202302312 37813488 PMC10561765

[18] Holmberg R , Robinson M , Gilbert SF , et al. TWEAK‐Fn14‐RelB signaling cascade promotes stem cell‐like features that contribute to post‐chemotherapy ovarian cancer relapse. Mol Cancer Res. 2023;21(2):170‐186. doi:10.1158/1541-7786.MCR-22-0486 36214671 PMC9890141

[19] Zhou M , Kang S , Xia Y , Zhang D , Chen W . ATP2C1 knockdown induces abnormal expressions of cytoskeletal and tight junction proteins mimicking Hailey‐Hailey disease. Indian J Dermatol Venereol Leprol. 2024;90(6):722‐730. doi:10.25259/IJDVL_853_2023 38841932

[20] Wen L , Miao Y , Fan Z , et al. Establishment of an efficient primary culture system for human hair follicle stem cells using the Rho‐associated protein kinase inhibitor Y‐27632. Front Cell Dev Biol. 2021;9:632882. doi:10.3389/fcell.2021.632882 33748117 PMC7973216

[21] Zou XY , Ding D , Zhan N , Liu XM , Pan C , Xia YM . Glyoxalase I is differentially expressed in cutaneous neoplasms and contributes to the progression of squamous cell carcinoma. J Invest Dermatol. 2015;135(2):589‐598. doi:10.1038/jid.2014.377 25184957

[22] Tierney MT , Polak L , Yang Y , et al. Vitamin A resolves lineage plasticity to orchestrate stem cell lineage choices. Science. 2024;383(6687):eadi7342. doi:10.1126/science.adi7342 38452090 PMC11177320

[23] Montazersaheb S , Fathi E , Farahzadi R . Cytokines and signaling pathways involved in differentiation potential of hematopoietic stem cells towards natural killer cells. Tissue Cell. 2021;70:101501. doi:10.1016/j.tice.2021.101501 33578272

[24] Wen M , Ying Y , Xiao X , et al. Ox40‐Cre‐mediated deletion of BRD4 reveals an unexpected phenotype of hair follicle stem cells in alopecia. JCI Insight. 2022;7(23):e164534. doi:10.1172/jci.insight.164534 36256455 PMC9746908

[25] Amoh Y , Hoffman RM . Hair follicle‐associated‐pluripotent (HAP) stem cells. Cell Cycle. 2017;16(22):2169‐2175. doi:10.1080/15384101.2017.1356513 28749199 PMC5736337

[26] Shen Q , Yu W , Fang Y , Yao M , Yang P . Beta‐catenin can induce hair follicle stem cell differentiation into transit‐amplifying cells through c‐myc activation. Tissue Cell. 2017;49(1):28‐34. doi:10.1016/j.tice.2016.12.005 28049551

[27] Short C , Zhong A , Xu J , et al. TWEAK/FN14 promotes profibrogenic pathway activation in Prominin‐1‐expressing hepatic progenitor cells in biliary atresia. Hepatology. 2023;77(5):1639‐1653. doi:10.1097/HEP.0000000000000026 36626628

[28] Chopra M , Brandl A , Siegmund D , et al. Blocking TWEAK‐Fn14 interaction inhibits hematopoietic stem cell transplantation‐induced intestinal cell death and reduces GVHD. Blood. 2015;126(4):437‐444. doi:10.1182/blood-2015-01-620583 26012567 PMC4536890

[29] Sarabia‐Sánchez MA , Robles‐Flores M . WNT signaling in stem cells: a look into the non‐canonical pathway. Stem Cell Rev Rep. 2024;20(1):52‐66. doi:10.1007/s12015-023-10610-5 37804416 PMC10799802

[30] He X , Cui Y , Li T , et al. PU.1 alleviates the inhibitory effects of cigarette smoke on endothelial progenitor cell function and lung‐homing through Wnt/beta‐catenin and CXCL12/CXCR4 pathways. Tob Induc Dis. 2024;22:10.18332/tid/174661. 10.18332/tid/174661 PMC10809061 38274000

[31] Wang X , Cheng D , Hu G , et al. Tumor necrosis factor (TNF) receptor expression determines keratinocyte fate upon stimulation with TNF‐like weak inducer of apoptosis. Mediators Inflamm. 2019;2019:2945083. doi:10.1155/2019/2945083 31885495 PMC6915140

[32] Hu G , Liang L , Liu Y , et al. TWEAK/Fn14 interaction confers aggressive properties to cutaneous squamous cell carcinoma. J Invest Dermatol. 2019;139(4):796‐806. doi:10.1016/j.jid.2018.09.035 30414907

[33] Cheng H , Xu M , Liu X , Zou X , Zhan N , Xia Y . TWEAK/Fn14 activation induces keratinocyte proliferation under psoriatic inflammation. Exp Dermatol. 2016;25(1):32‐37. doi:10.1111/exd.12820 26264384

[34] Ba H , Li B , Li X , et al. Transmembrane tumor necrosis factor‐α promotes the recruitment of MDSCs to tumor tissue by upregulating CXCR4 expression via TNFR2. Int Immunopharmacol. 2017;44:143‐152. doi:10.1016/j.intimp.2016.12.028 28092866

[35] Kuo YC , Au HK , Hsu JL , et al. IGF‐1R promotes symmetric self‐renewal and migration of alkaline phosphatase + germ stem cells through HIF‐2α‐OCT4/CXCR4 loop under hypoxia. Stem Cell Rep. 2018;10(2):524‐537. doi:10.1016/j.stemcr.2017.12.003 PMC5830933 29307582

[36] Wang Q , Zhang F , Hong Y . Blocking of autocrine IGF‐1 reduces viability of human umbilical cord mesenchymal stem cells via inhibition of the Akt/Gsk‐3β signaling pathway. Mol Med Rep. 2018;17(3):4681‐4687. doi:10.3892/mmr.2018.8445 29344668

[37] Xia Y , Chen J , Ding J , Zhang J , Chen H . IGF1‐ and BM‐MSC‐incorporating collagen‐chitosan scaffolds promote wound healing and hair follicle regeneration. Am J Transl Res. 2020;12(10):6264‐6276. PMID: 33194028. 33194028 PMC7653568

