# Supplementary material for: TWEAK regulates the functions of hair follicle stem cells via the Fn14‐Wnt/β‐catenin‐CXCR4 signalling axis
Source: Wound Repair Regen. 2025 May 5;33(3):e70032. doi: 10.1111/wrr.70032 (PMC12053109; doi:10.1111/wrr.70032)
Supplement: Supplementary file 1 — Figure S1. TWEAK, Fn14 and HFSC markers are highly expressed around cutaneous wounds in mice. (A) The expressions of K15, integrin β1 and Lgr5 were prominent in the wound or normal areas. (B) Both TWEAK and Fn14 were highly expressed in these areas with different patterns. Data are from three independent experiments. Representative images are shown. Scale bar = 30 μm. [file WRR-33-0-s002.pdf]

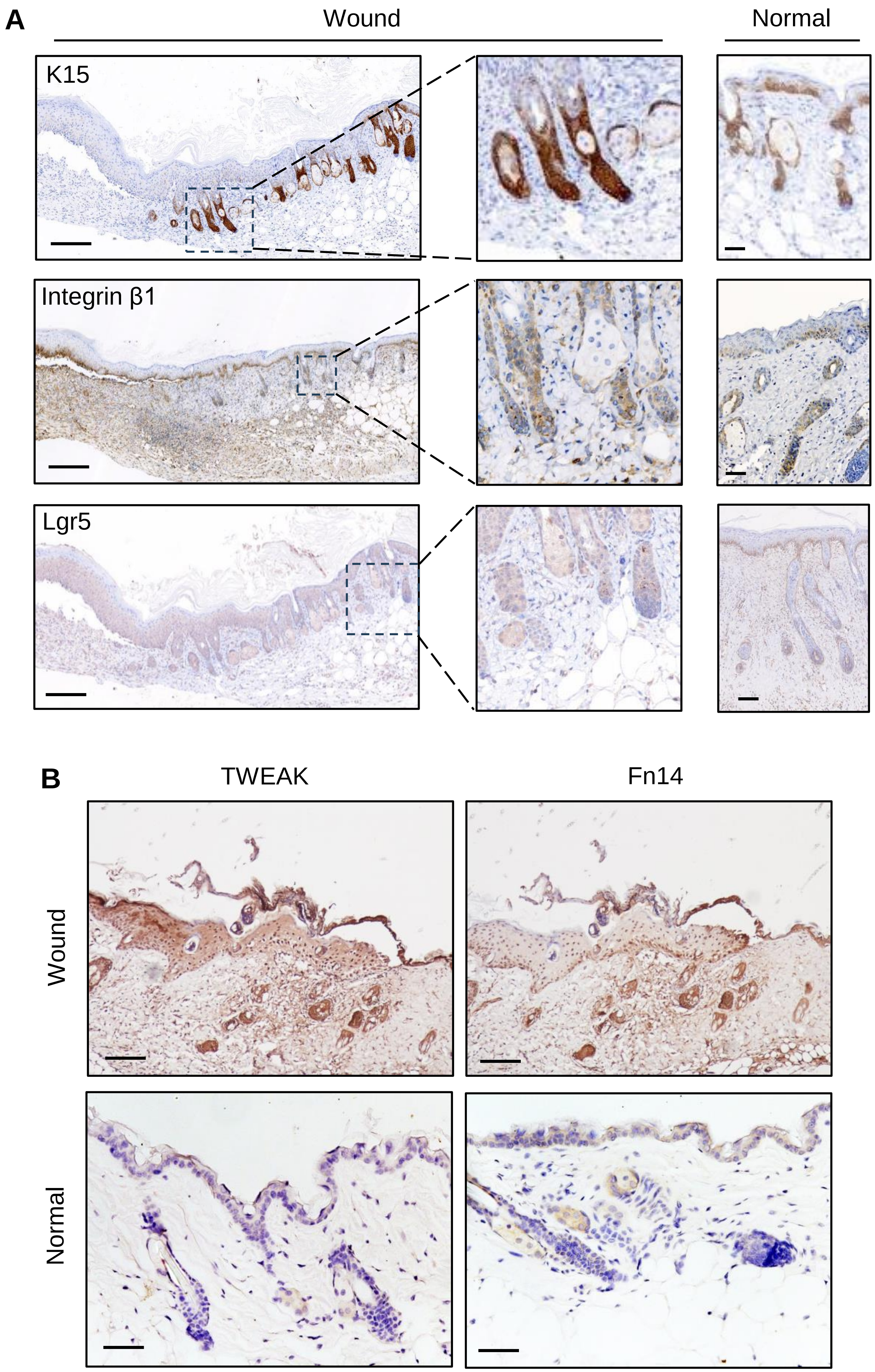
A
Wound
Normal
K15
Integrin β1
Lgr5
B
TWEAK
Fn14
Wound
Normal

Supplementary Figure S1. TWEAK, Fn14, and HFSC markers are highly expressed around cutaneous wounds in mice. (**A**) The expressions of K15, integrin β1, and Lgr5 were prominent in the wound or normal areas. (**B**) Both TWEAK and Fn14 were highly expressed in these areas with different patterns. Data are from three independent experiments. Representative images are shown. Scale bar = 30 μm.
