# Supplementary material for: TWEAK regulates the functions of hair follicle stem cells via the Fn14‐Wnt/β‐catenin‐CXCR4 signalling axis
Source: Wound Repair Regen. 2025 May 5;33(3):e70032. doi: 10.1111/wrr.70032 (PMC12053109; doi:10.1111/wrr.70032)
Supplement: Supplementary file 2 — Table S1. Antibodies used for immunohistochemistry or Western blotting. Table S2. Primers used for qRT‐PCR analysis. [file WRR-33-0-s001.docx]

**Supplementary Table S1.** Antibodies used for immunohistochemistry or Western blotting

| Antibody | Company | Catalog Number | Application |
| --- | --- | --- | --- |
| β-actin | Abcam | ab124964 | WB |
| β-catenin | Abcam | ab305261 | WB |
| CD34 | Abcam | ab81289 | IHC |
| CXCR4 | Abcam | ab216548 | IF |
| CXCR4 | CST | 64837 | WB |
| Fn14 | Abcam | ab313079 | IF |
| Fn14 | CST | 4403 | IHC, WB |
| GSK3β | Abcam | ab32391 | WB |
| IGFR | CST | 9750 | WB |
| Integrin β1 | Abcam | ab202643 | IF |
| Integrin β1 | Abcam | ab179471 | WB |
| K15 | Abcam | ab194065 | IF |
| K15 | Abcam | ab52816 | WB |
| K19 | CST | 12434 | IHC, WB |
| TNFR2 | CST | 72337 | WB |
| TNFR2 | CST | 99347 | IF |
| Wnt5a | Abcam | ab235966 | WB |

Abbreviations: IF, immunofluorescence; IHC, immunohistochemistry; WB, Western blotting; CST, Cell Signaling Technology (company)

**Supplementary Table S2.** Primers used for qRT-PCR analysis

| Gene | Species | Forward (5’ to 3’) | Reverse (5’ to 3’) |
| --- | --- | --- | --- |
| *BFGF* | Human | GCTGTACTGCAAAAACGGGG | AGCCAGGTAACGGTTAGCAC |
| *CTNNB* | Human | CTGAGGAGCAGCTTCAGTCC | ATTGCACGTGTGGCAAGTTC |
| *CXCR4* | Human | ATCAGTCTGGACCGCTACCT | CCACCTTTTCAGCCAACAGC |
| *EGF* | Human | AGAGGGAGAGGATGCCACAT | ACAAACCAAGGTTGAGGGCA |
| *Fn14* | Human | CTCTGAGCCTGACCTTCGTG | GGGGGCACATTGTCACTGGA |
| *GAPDH* | Human | GCACCGTCAAGGCTGAGAAC | TGGTGAAGACGCCAGTGGA |
| *GSK3B* | Human | GCAGCAAGGTGACAACAGTG | AAGAGTGCAGGTGTGTCTCG |
| *IGFR* | Human | GGCTTTGCCCCCTTTCTTTG | TCGTCGGCCTCCATTTTCAA |
| *NGF* | Human | GCGCAGCGAGTTTTGGC | GGATGGGATGATGACCGCTT |
| *TGF-β* | Human | CGGATCTCTTCCTGCTCGAC | CACAGTAGTAGGCGGCGTAG |
| *TNFR2* | Human | GGGATAAAGGAGAAGGCATGAA | TCTCCCACCCTCCAATAGTT |
| *VEGF* | Human | AGGCCAGCACATAGGAGAGA | ACGCGAGTCTGTGTTTTTGC |
| *WNT5A* | Human | CCAGGAGTTGCTTTGGGGAT | CCTCCGACGTCTTGTGACAT |

Note: *BFGF* codes bFGF protein; *CTNNB* codes β-catenin protein.
